# Arginine Vasotocin Preprohormone Is Expressed in Surprising Regions of the Teleost Forebrain

**DOI:** 10.3389/fendo.2017.00195

**Published:** 2017-08-14

**Authors:** Mariana Rodriguez-Santiago, Jessica Nguyen, Lin S. Winton, Chelsea A. Weitekamp, Hans A. Hofmann

**Affiliations:** ^1^Institute for Neuroscience, The University of Texas at Austin, Austin, TX, United States; ^2^Department of Integrative Biology, The University of Texas at Austin, Austin, TX, United States; ^3^Institute for Cell and Molecular Biology, The University of Texas at Austin, Austin, TX, United States

**Keywords:** nonapeptide, arginine vasopressin, arginine vasotocin, behavior, preoptic area, amygdala, hippocampus

## Abstract

Nonapeptides play a fundamental role in the regulation of social behavior, among numerous other functions. In particular, arginine vasopressin and its non-mammalian homolog, arginine vasotocin (AVT), have been implicated in regulating affiliative, reproductive, and aggressive behavior in many vertebrate species. Where these nonapeptides are synthesized in the brain has been studied extensively in most vertebrate lineages. While several hypothalamic and forebrain populations of vasopressinergic neurons have been described in amniotes, the consensus suggests that the expression of AVT in the brain of teleost fish is limited to the hypothalamus, specifically the preoptic area (POA) and the anterior tuberal nucleus (putative homolog of the mammalian ventromedial hypothalamus). However, as most studies in teleosts have focused on the POA, there may be an ascertainment bias. Here, we revisit the distribution of AVT preprohormone mRNA across the dorsal and ventral telencephalon of a highly social African cichlid fish. We first use *in situ* hybridization to map the distribution of AVT preprohormone mRNA across the telencephalon. We then use quantitative real-time polymerase chain reaction to assay AVT expression in the dorsomedial telencephalon, the putative homolog of the mammalian basolateral amygdala. We find evidence for AVT preprohormone mRNA in regions previously not associated with the expression of this nonapeptide, including the putative homologs of the mammalian extended amygdala, hippocampus, striatum, and septum. In addition, AVT preprohormone mRNA expression within the basolateral amygdala homolog differs across social contexts, suggesting a possible role in behavioral regulation. We conclude that the surprising presence of AVT preprohormone mRNA within dorsal and medial telencephalic regions warrants a closer examination of possible AVT synthesis locations in teleost fish, and that these may be more similar to what is observed in mammals and birds.

## Introduction

A fundamental aspect of studying animal physiology and behavior is understanding the pathways and mechanisms by which they are regulated. Many studies have focused on understanding how certain neurochemicals, such as neurotransmitters or neuromodulators, influence behavior. One such family of neurochemicals, a class of nine amino acid molecules known as nonapeptides, is of particular interest. Nonapeptides are highly conserved across vertebrates and play crucial roles in numerous physiological functions and behaviors (1). Their exact effects vary widely between species for reasons that are not fully clear, making them the subject of studies spanning taxa, sexes, social contexts, brain regions, and scientific fields.

One of the nonapeptides, arginine vasopressin (AVP; also known as antidiuretic hormone, ADH), is of particular interest in the study of social behavior across animals. AVP is a highly conserved nonapeptide that has a wide range of modulatory effects across vertebrates (2). Most vertebrate classes possess the ancestral nine amino acid peptide form, arginine vasotocin (AVT; AVP has a phenylalanine substitution of isoleucine in position 3) (3). Originally identified for its role in osmoregulation, cardiovascular function, and stress hormone release (4–6), AVP/T has also been shown to play a key role in modulating social behavior such as courtship and aggressive behavior in fish (7–9), amphibians (10–12), birds (13–16), and in mammals (17–19). AVP/T has also been shown to modulate territoriality and space use [reviewed in Ref. (20)] and alternative reproductive phenotypes in teleost fish (21–29). These effects are mediated by sex, social context, and the neural expression of the nonapeptide and its receptors (2, 3).

**Figure 1:**
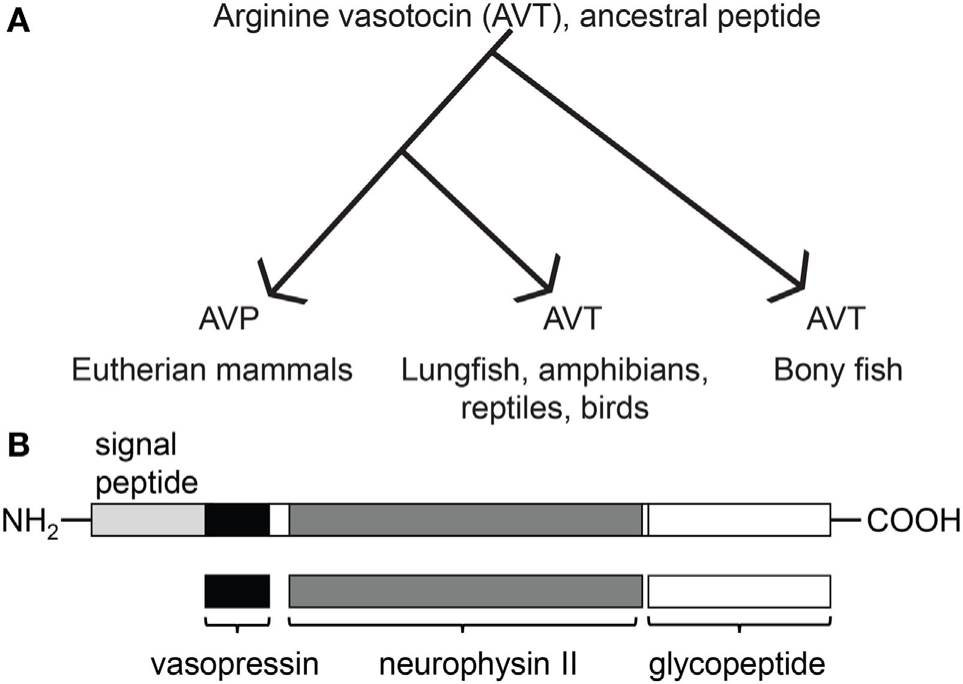
Evolutionary relationship between arginine vasotocin (AVT) and arginine vasopressin (AVP), and the composition of AVT/AVP prohormone and its products. **(A)** Evolution of the vertebrate AVT nonapeptide family [originally modeled after Acher and Chauvet 1995 and adapted from Ref. (31)]. **(B)** Prepropressophysin undergoes post-translational modifications and yields three peptides, namely vasopressin, neurophysin II, and a glycopeptide [based on data derived from Ref. (32)].

AVP/T is synthesized in magnocellular neurons of the hypothalamus in animals and is produced from prohormones that also encode a carrier protein, neurophysin. There are two types of neurophysin: the prohormone proxyphysin that is hydrolyzed to oxytocin and neurophysin I, and the prohormone propressophysin that is hydrolyzed to vasopressin and neurophysin II, in addition to a short glycopeptide (Figure 1). Studies previously done in mammals have shown that these distinct neurophysins may be essential for the implementation of hormonal activity (30). The axon terminals of these hypothalamic neurons extend to the neurohypophysis, where the secretions of these neurosecretory cells are picked up by the circulatory system and transported to target organs.

In the brain, AVP/T exerts its effects in particular regions by binding to distinct receptors. The expression of these receptors differs across tissues and by function (33, 34). For example, the AVP/T receptor subtype, V1a, has been shown to regulate sex and species differences in many social behaviors in mammals, birds, amphibians, and fish (24, 35–37). For teleosts in particular, AVT receptors consist of one V2-type and two V1a types (V1a1 and V1a2) (38–40). The distributions of these receptors are widespread throughout the brain and are found in regions of interest for social regulation, such as the olfactory bulb (OB), telencephalic areas, POA, hypothalamus, midbrain sensory regions, and hindbrain regions important for social approach responses (41, 42).

AVP/T cell bodies are found in the preoptic area-anterior hypothalamus (POA-AH) complex, an integration center that also regulates numerous physiological and hormonal processes through the pituitary gland (16, 23, 43–48). AVP/T peptides are produced by populations of magnocellular and parvocellular neurons within this POA-AH complex. In amniotes, these magnocellular neurons are found in the supraoptic nucleus (SON) of the hypothalamus, while parvocellular neuron populations are found in the paraventricular nucleus (PVN) of the hypothalamus (3, 47). In fish and amphibians, AVT in these magnocellular and parvocellular neuronal populations are found in the POA and AH. These cell groups project to the neurohypophysis, where AVP/T exerts a wide range of peripheral effects (31). Previous studies have used immunohistochemical (IHC) techniques to label immunoreactivity of AVP/T protein product, or *in situ* hybridization (ISH) to label AVP/T preprohormone mRNA across the brain. Table 1 provides a summary of the brain regions where AVP/T has been found, along with the technique used to map either AVP/T protein product or label AVP/T preprohormone mRNA in the respective studies. In general, amniotes have similar patterns of AVT expression throughout the forebrain. In teleosts, however, AVT-containing neurons have been shown to be localized to the POA region.

**Table 1:** Presence of forebrain arginine vasotocin/arginine vasopressin across vertebrates.

Class	Brain regions	Species	Study	Methods
Fish	Diencephalon: Preoptic area	*Anguilla anguilla*	Olivereau et al. (49)	IHC
		*Astatotilapia burtoni*	Greenwood et al. (21)	*In situ* hybridization (ISH)
		*Carassius auratus*	Reaves and Hayward (50)	
		*Halichoeres trimaculatus*	Hur et al. (51)	qPCR
		*Oncorhynchus keta*	Ota et al. (52)	ISH, IHC
		*Oncorhynchus masou*	Ota et al. (28, 53)	ISH, IHC
		*Oncorynchus mykiss*	Gilchriest et al. (54)	ISH
		*Poecilia latipinna*	Batten et al. (55)	IHC
		*Protopterus aethiopicus*	Goossens et al. (56)	IHC
		*Prochthys notatus*	Goodson and Bass (22, 23)	IHC
		*Salmo gairdneri*	van den Dungen et al. (57)	IHC
		*Scyliorhinus caniculus*	Vallarino et al. (58)	IHC
		*Thalassoma bifasciatum*	Godwin et al. (59)	ISH
		*Xiphophorus maculatus*	Schreibman and Halpern (60)	IHC

Amphibians	Pallial telencephalon	*Pleurodeles waltlii*	Gonzalez and Smeets (61, 62)	IHC
	Subpallial telencephalon	*Rana catesbeiana*	Boyd et al. (63); Gonzalez and Smeets (61, 62); Mathieson (64)	IHC
		*Rana ridibunda*	Gonzalez and Smeets (61, 62)	IHC
		*Rana sylvatica*	Mathieson (64)	IHC
		*Taricha granulosa*	Lowry et al. (65); Lowry et al. (45)	ISH, IHC
		*Xenopus laevis*	Gonzalez and Smeets (61, 62)	IHC
	Diencephalon: BNST and POA	*Bufo japonicus*	Jokura and Urano (66)	IHC
		*Pseudemys scripta*	Smeets et al. (67)	IHC
		*Rana catesbeiana*	Boyd et al. (63)	IHC
		*Taricha granulosa*	Lowry et al. (45)	ISH, IHC
		*Typhlonectes compressicauda*	Gonzales and Smeets (68)	IHC
		*Typhlonectes natans*	Hilscher-Conklin et al. (69)	IHC
		*Xenopus laevis*	Gonzalez and Smeets (61, 62)	IHC

Reptiles	Subpallial telencephalon	*Anolis carolinensis*	Propper et al. (70)	IHC
		*Pseudemys scripta elegans*	Smeets et al. (71)	IHC
		*Python regius*	Smeets et al. (71)	IHC
		*Gekko gecko*	Stoll and Voorn (72); Thepen et al. (73)	IHC
	Diencephalon: POA, thalamic regions	*Anolis carolinensis*	Propper et al. (70)	IHC
		*Gekko gecko*	Stoll and Voorn (72); Thepen et al. (73)	IHC
		*Lacerta muralis*	Bons (74)	IHC
		*Mauremys caspica*	Fernandez-Llebrez et al. (75)	IHC
		*Natrix maura*	Fernandez-Llebrez et al. (75)	IHC
		*Pseudemys scripta elegans*	Smeets et al. (71)	IHC
		*Python regius*	Smeets et al. (71); Smeets et al. (67)	IHC

Birds	Subpallial telencephalon	*Coturnix japonica*	Aste et al. (76)	ISH
		*Gallus domesticus*	Aste et al. (76); Jurkevich et al. (77)	ISH, IHC
		*Junco hyemalis*	Panzica et al. (78)	IHC
		*Serinus canaria*	Kiss et al. (79)	IHC
		*Taeniopygia guttata*	Voorhuis and de Kloet (80)	IHC
	Diencephalon: POA, thalamic regions	*Columba livia*	Berk et al. (81)	IHC
		*Coturnix japonica*	Bons (82); Panzica et al. (83)	IHC

		*Serinus canaria*	Kiss et al. (79)	IHC
		*Taeniopygia guttata*	Voorhuis and de Kloet (80)	IHC

Mammals	Subpallial telencephalon	*Felis catus*	Caverson et al. (84)	
		*Macaca fascicularis*	Caffe et al. (85)	IHC
		*Mesocricetus auratus*	Dubois-Dauphin et al. (86)	IHC
		*Mus musculus*	Castel and Morris (87)	IHC
		*Rattus norvegicus*	Rhodes et al. (88); DeVries et al. (89); van Leeuwen et al. (90); Urban et al. (91); Wang et al. (92); Planas et al. (93)	IHC, ISH
		*Sus scrofa*	van Eerdenburg et al. (94)	IHC
	Diencephalon: POA, hypothalamic regions	*Cavia porcetella*	Dubois-Dauphin et al. (86)	IHC
		*Felis catus*	Caverson et al. (84)	
		*Jaculus orientalis*	Lakhdar-Ghazal et al. (95)	IHC
		*Macaca fascicularis*	Caffe et al. (85)	IHC
		*Meriones unguiculatus*	Wu and Shen (96)	IHC
		*Mus musculus*	Castel and Morris (87)	IHC
		*Rattus norvegicus*	Rhodes et al. (88); DeVries et al. (89); Dobie et al. (97); Miller et al. (98); Miller et al. (99); Brot et al. (100); Szot and Dorsa (101); Szot and Dorsa (102)	IHC, ISH

Tetrapod vertebrates exhibit additional anatomical characteristics that remain largely conserved. AVP is produced in neurons of the bed nucleus of the stria terminalis and the medial amygdala, and projections extend to the lateral septum, nucleus accumbens, amygdala, and periaqueductal gray (PAG) (47, 103, 104). These circuits are particularly important for social behavior, such as mate affiliation, nest defense, and parental care of offspring (92, 105–107). Putative teleost homologs of these regions also contain AVT fiber innervation, though these fibers are generally thought to originate in the POA (22, 55). AVP/T fibers are located throughout the brain in jawed vertebrates, likely conserved for at least 500 million years, including the POA, anterior and lateral hypothalamic areas, midbrain tegmentum, PAG, isthmal structures (i.e., locus coeruleus), and viscerosensory areas of the caudal medulla (3).

In the teleost POA, the magnocellular and gigantocellular AVT neuron populations are hypothesized to be homologous to the supraoptic nucleus in tetrapods based on colocalization with corticotropin-releasing hormone-producing neurons and expression of the Nurr1 receptor, while the parvocellular cell group is the putative homolog of the PVN of the mammalian POA (47, 49, 108, 109). AVT appears to be limited to the POA (1). Weaker expression also appears in the anterior tuberal nucleus of the hypothalamus [aTn; (21, 23)], the putative teleost homolog of the mammalian ventromedial hypothalamus [VMH; (110, 111)]. As in tetrapods, AVT is found in the parvocellular, magnocellular, and gigantocellular neuron groups, which are distinguished by soma size and location, with gigantocellular populations being found most caudally. These AVT neurons have been shown to project to the posterior pituitary through the preoptico-hypophysial tract as well as various regions in the ventral telencephalon and ventral thalamus (23, 112). Overall, the expression of AVT preprohormone mRNA and peptide seems to be fairly conserved across vertebrates. There might be an ascertainment bias as most studies only report on the POA and/or used IHC methods to map AVT-positive neurons, which may not be sensitive enough to detect low levels of peptide expression in other brain regions [but see Ref. (21, 51, 59, 113)].

Importantly, AVP/T has been shown to be socially regulated [see Ref. (3, 20) for reviews]. For example, non-monogamous male Montane voles have fewer V1a receptors in the ventral pallidum compared to monogamous Prairie voles, and the induction of these receptors in the Montane voles *via* viral vector gene transfer yields pair bonding behavior similar to Prairie voles (114). White-throated male sparrows (*Zonotrichia albicollis*) have more AVT expression in the medial portion of the BNST and in a subdivision of the caudal lateral septum compared to tan-striped male sparrows. This neural AVT expression is associated with aggression, since white-striped males defend their territories more vigorously and intrude into other territories more often than their tan-striped male counterparts (115). Research in teleosts suggests that AVT preprohormone mRNA levels might be more reliable indicators of social status than the number or size of AVT-positive neurons (as determined by immunohistochemistry). In Burton’s Mouthbrooder cichlid, *Astatotilapia burtoni*, socially dominant males exhibit higher levels of AVT expression than subordinate males in gigantocellular nucleus of the preoptic area, whereas the inverse was found in the parvocellular preoptic nucleus (21). The number or size of AVT-immune-reactive (ir) neurons was, however, not correlated with behavior (126). Similarly, in the sex-changing Bluehead wrasse, *Thalassoma bifasciatum*, preoptic AVT mRNA levels predicts male behavior robustly, while AVT-ir neuron size does not (59). These examples illustrate the role AVP/T plays in modulating social behavior across species, and how these effects are not just sex- and context-specific but also brain region-specific.

The majority of studies that examine the expression and distribution of either AVT preprohormone mRNA or the AVT peptide in teleost fish have primarily focused on the POA. These studies utilize quantitative real-time polymerase chain reaction (qPCR), immunohistochemistry, immunocytochemistry, or radioactive ISH to quantify mRNA and/or protein expression (for more information regarding these methods see Table 2). In the present study, we revisit the neural distribution of AVT nonapeptide expression, in particular expanding on the existing knowledge of its mRNA distribution within the forebrain of a highly social cichlid fish. We first used ISH to examine whether the AVT preprohormone mRNA is expressed in pallial and subpallial regions of the telencephalon of *A. burtoni*. In a second experiment, we used qPCR to ask whether AVT preprohormone mRNA expression in pallial area Dm, the putative homolog of the mammalian basolateral amygdala, is modulated by social context. We provide evidence of AVT preprohormone mRNA expression in forebrain regions never previously reported to contain nonapeptides in teleost fish. Furthermore, our results suggest that AVT preprohormone mRNA expression in the putative homolog of the mammalian basolateral amygdala can be regulated by social context.

**Table 2:** Differences between methodological techniques.

Technique	How does it work?	What is measured and visualized?	Advantages of each method
Quantitative real-time polymerase chain reaction (qPCR)	Binds cDNA (complementary DNA, after reverse transcription of mRNA) with a light-emitting molecule	Amplified cDNA	Quantitative

*In situ* hybridization (ISH)	Binds nucleic acid strands complementary to the mRNA of interest which is labeled with a chromophore or radioisotope	mRNA, fluorophore, or silver grains	Spatial resolution

Immunohistochemistry (IHC)	Uses an antibody that specifically binds a protein of interest for visualization in sectioned tissues, these antibodies are visible under fluorescence or brightfield microscopy when bound to a fluorophore or chromophore	Protein, cells or fibers	Spatial resolution

## Materials and Methods

### Study 1: AVT Distribution in the Cichlid Forebrain

#### Animals

The African cichlid fish, *Astatotilapia burtoni* (Burton’s Mouthbrooder), has become an important model system for the study of social neuroscience. Males of this species can be one of two phenotypes—dominant or subordinate—and this reversible phenotype depends on the immediate social context. Dominant males are highly territorial, aggressive, and reproductively active while subordinate males are non-reproductive and non-territorial. *A. burtoni* descended from a wild-caught stock population were kept in aquaria under naturalistic environmental conditions and stable naturalistic communities as previously described (116). The animals used for mapping the distribution of AVT with ISH were the same as those used in a previous study (42). All work was carried out in compliance with the Institutional Animal Care and Use Committee at the University of Texas at Austin.

#### *In Situ* Hybridization

**Figure 2:**
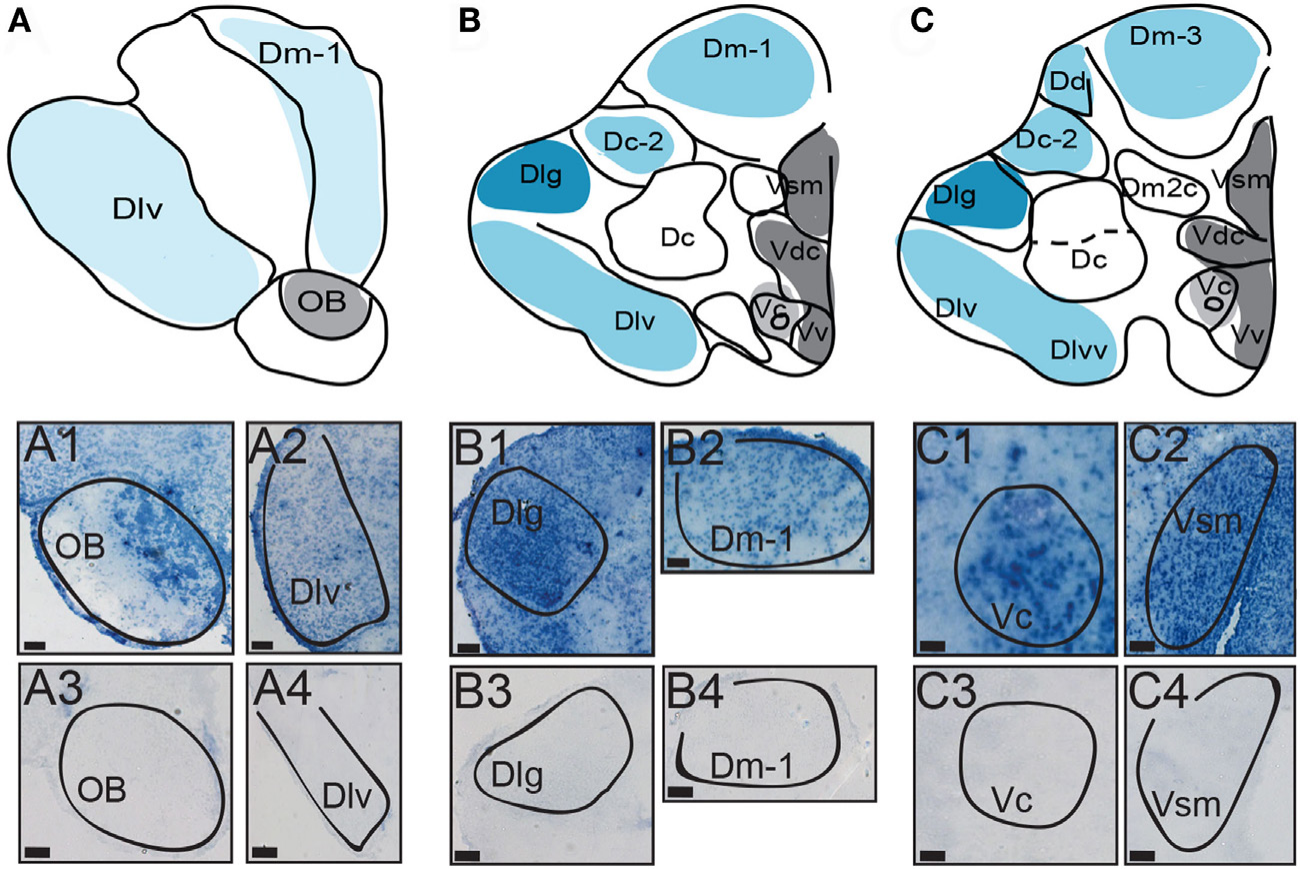
Distribution of AVT preprohormone mRNA in the telencephalon. **(A–C)** The first row represents a template marked with the distribution of AVT preprohormone mRNA. mRNA is shown as shading on the representative template, and the degree of shading corresponds to the qualitative density of expression. Micrographs show AVT preprohormone mRNA in the olfactory bulb (OB; A1), in the ventrolateral part of D (Dlv; A2), the granular region of D (Dlg; B1), a subregion of the medial part of D (Dm-1; B2), the central part of V (Vc; C1), and in the medial part of Vs (Vsm, C2). The sense controls show a lack of AVT preprohormone mRNA signal in the OB (A3), Dlv (A4), Dlg (B3), Dm-1 (B4), Vc (C3), and Vsm (C4). All scale bars are shown at 20 µm.

Brains from dominant and subordinate males and females were rapidly dissected and fresh frozen in OCT compound (Tissue-Tek, USA) on dry ice, and stored at −80°C. Brains were subsequently sectioned and stored until processing for ISH as previously described (116). Due to regions of high sequence similarity in the coding regions between neuropeptides and receptors used in the original study (42), the probe for AVT was designed to identify the 3′ untranslated region. The template used to make the AVT probe was 378 bp in length (21). Experimental slides were exposed to anti-sense fluorescein-labeled probe, whereas control slides were incubated with sense fluorescein-labeled probe (Figure 2). After the overnight hybridization, slides were processed for detection of mRNA by non-radioactive, non-fluorescent detection. Sections were washed in a series of 0.2x SSC washes at 65°C and equilibrated in 150 mM NaCl/100 mM Tris (pH 7.5) at room temperature before incubation in 1:1,000 anti-fluorescein-alkaline phosphatase Fab fragments (Roche) in 0.05% Tween 20/PBS for 2 h at room temperature. Sections were then washed in 150 mM NaCl/100 mM Tris (pH 7.5). Chromogenic product was formed using BM Purple (Roche) at room temperature until desired darkness was achieved and was terminated simultaneously for all slides within a gene group. Slides were then washed, dehydrated in an ethanol series ending in xylene, and cover-slipped with Permount (Fisher Scientific). These slides were previously used in Ref. (42) to examine the distribution of AVT and isotocin receptor in *A. burtoni*.

#### Microscopy

Micrographs were captured and processed as previously detailed (42). Brightfield optics were used to visualize staining throughout the brain at low (5×) and high magnification (10×). Photographs were taken with a digital camera (AxioCam MRc, Zeiss) attached to a Zeiss AxioImager.A1 AX10 microscope using the AxioVision (Zeiss) image acquisition and processing software. Images were compiled and brightness-enhanced in Adobe Photoshop.

### Study 2: AVT Expression Variation in Dm in Socially Relevant Contexts

#### Animals

*A. burtoni* descended from a wild-caught stock population were kept in stable naturalistic communities, as described (117) until they were transferred into the experimental conditions. These animals were the same as those used in a previous study (118). All work was carried out in compliance with the Institutional Animal Care and Use Committee at the University of Texas at Austin.

#### Behavior

Animals were placed in experimental tanks which had one territorial male and two non-reproductive females [as described in Ref. (118)]. Focal males were tested in one of three social contexts; namely (1) a Reproductive Context, in which an adjacent tank contained one gravid and two non-reproductive females, (2) a Familiar Neighbor context, in which the adjacent tank contained one size-matched territorial male and two non-reproductive females, and (3) a Neutral Stimulus context that contained three non-reproductive females. Non-reproductive females were stripped of their brood immediately before placement in each tank, ensuring that they would remain non-reproductive for the duration of the study (119). Males were killed by rapid cervical transection and brains were flash frozen in O.C.T. (Tissue-Tek; Fisher Scientific Co., Pittsburgh, PA, USA) and stored at −80°C.

#### Quantitative Real-time Polymerase Chain Reaction

Brains were sectioned on a cryostat in the transverse plane at 300 µm. A 300 µm diameter sample corer tool (Fine Science Tools, Foster City, CA) was used to micro-dissect the Dm-1. Two micro-dissected punches (left and right hemisphere) were taken from a single brain slice and stored in DNA/RNA Shield (Zymo Research, Irvine, CA, USA) at −80°C until processing. ZR BashingBeads (Zymo Research) were added to samples suspended in DNA/RNA Shield for tissue homogenization before RNA extraction. Proteinase K digestion was done for 2 h at 55°C to lyse tissue. Total RNA was then extracted in accordance with the protocol for the Quick-RNA MicroPrep kit (Zymo Research, Irvine, CA, USA). RNA samples were treated with DNase (Zymo) during isolation procedure to prevent DNA contamination. The GoScript Reverse Transcription System (Promega Corporation, Madison, WI, USA) was used to reverse transcribe RNA to cDNA.

Quantitative real-time polymerase chain reaction was used to measure the mRNA levels of AVT preprohormone and the primers were designed to flank exon-exon boundaries (AVT forward: 5′-AGGCAGGAGGGAGATCCTGT; AVT reverse: 5′-CAGGCAGTCAGAGTCCACCAT. 18S forward: 5′-CCCTTCAAACCCTCTTACCC; 18S reverse: 5′-CCACCGCTAAGAGTCGTATT). Target gene expression was measured in triplicate in the ViiA™ 7 Real-time PCR System (Applied Biosystems, Foster City, CA, USA) using GoTaq qPCR Master Mix (Promega). Amplification efficiency for the primer pair was determined using standard curves made from serial dilutions of cDNA.

#### Statistical Analyses

Statistical tests were performed using R v. 3.1.0. We used the R package mcmc.qpcr to determine relative gene expression for each sample. 18S was used as a control gene, and other target genes measured within the same region were included in the normalization analysis. This package analyzes qPCR data using generalized linear mixed models based on lognormal Poisson error distribution, fitted using Markov chain Monte Carlo statistical methods (120).

## Results

### *In Situ* Hybridization of AVT Preprohormone mRNA across the Pallium and Subpallium

**Figure 3:**
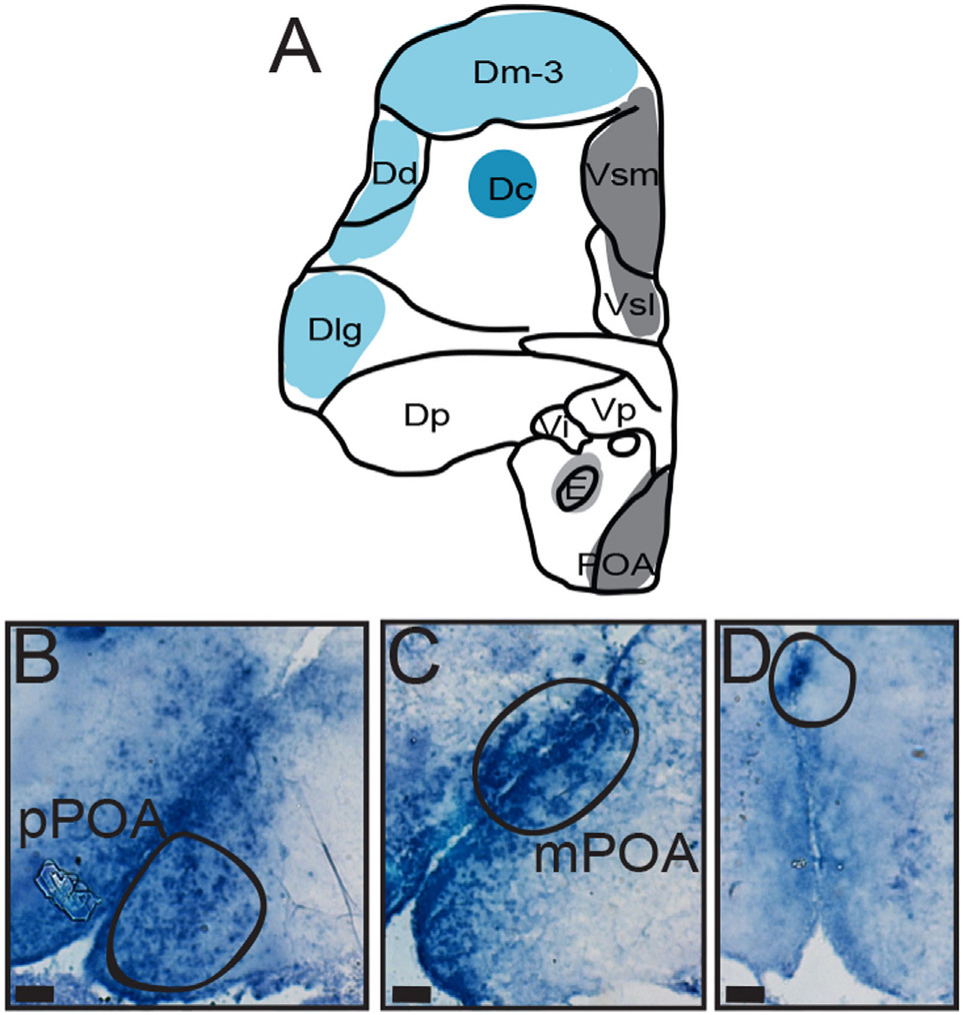
Distribution of AVT preprohormone mRNA in the preoptic area (POA) of *A. burtoni*. **(A)** A template marked with the distribution of AVT preprohormone mRNA (shading). The degree of shading corresponds to the qualitative density of expression. **(B)** Micrograph shows AVT preprohormone mRNA in the parvocellular population of the POA. **(C)** Micrograph shows AVT preprohormone mRNA in the magnocellular population of the POA. **(D)** Micrograph shows AVT preprohormone mRNA in the gigantocellular population of the POA. All scale bars are shown at 20 µm.

**Figure 4:**
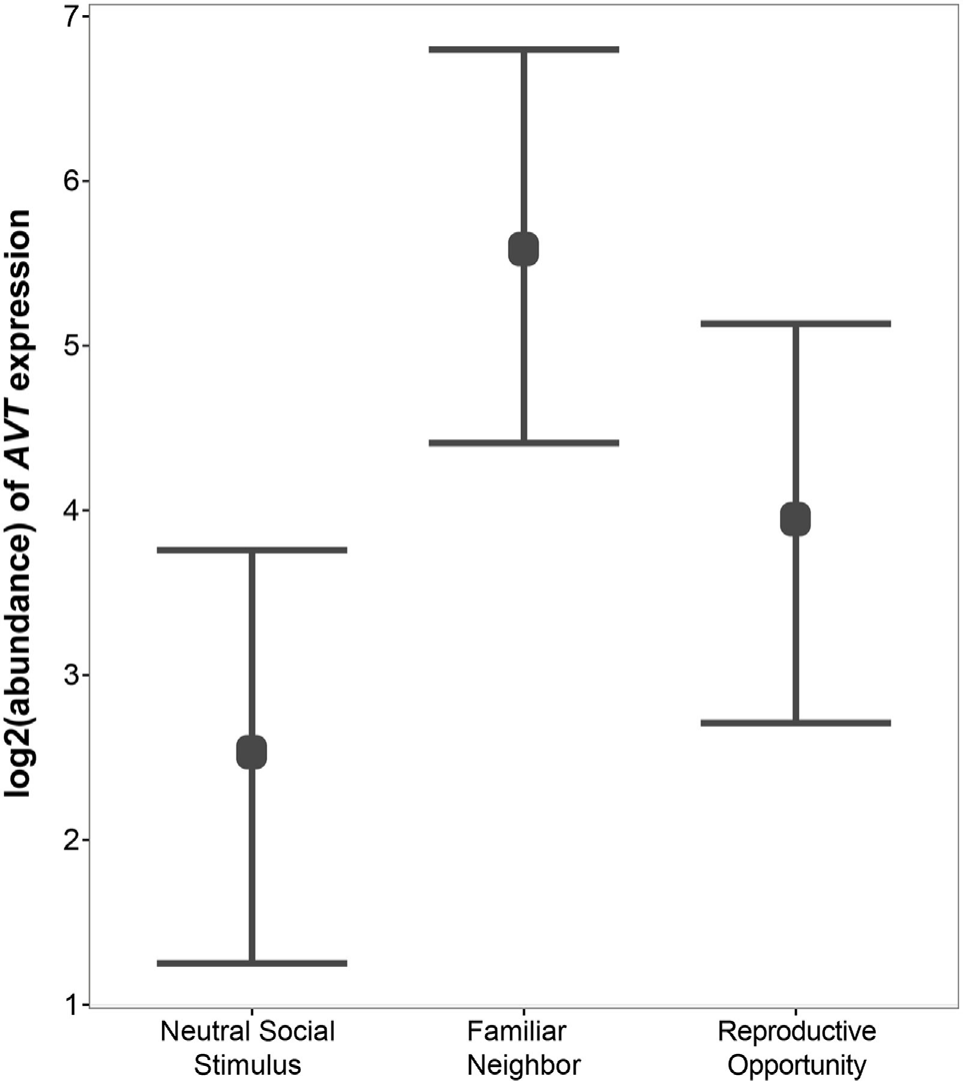
Relative gene expression of AVT preprohormone mRNA across social contexts. AVT expression was highest in individuals engaged in Familiar Neighbor social context, and is generally higher in contexts in which the social stimulus is not neutral, such as Familiar Neighbor and Reproductive Opportunity contexts, as compared to a context with a Neutral Social Stimulus.

We first describe the distribution of AVT preprohormone mRNA throughout the *A. burtoni* pallium and subpallium using ISH. In Figures 3 and 4, we present a distribution maps along with photomicrographs of representative brain areas for AVT expression in the *A. burtoni* brain. For each representative section of the map, the teleost nomenclature is displayed along with the preprohormone distribution. The degree of shading represents the approximate density of mRNA expression in that brain region. Pallial regions are colored in shades of blue while subpallial regions are colored in shades of gray. The general patterns are qualitatively independent of reproductive or social status and similar in males and females. Control slides hybridized with sense probes showed no specific signal (Figure 2).

Robust expression of AVT preprohormone mRNA is seen throughout the *A. burtoni* pallium. AVT preprohormone mRNA is present in the central, medial and lateral parts of the pallium (Dc, Dm, and Dl, respectively, Figure 2). The ventral subregion of Dl (Dlv) has mild staining of AVT preprohormone mRNA (Figure 2, A2), while the granular part of Dl (Dlg) has darker staining (Figure 2, B1). AVT preprohormone mRNA is present across all subdivision of the Dm (Dm-1,2,3) but has lighter stain in the Dm-1 subdivision (Figure 2, B2). The Dc-2 subdivision of the Dc telencephalon also shows light staining of AVT preprohormone mRNA, which is absent from the Dc (Figure 2B). In general, AVT expression becomes more robust in more caudal sections of these pallial regions.

There is robust AVT expression within the OB and subpallium as well as in the granule cell layer of the OB (Figure 2, A1), while preprohormone mRNA is predominantly absent from the glomeruli region. Ventral, central, and supracommissural parts (Vv, Vc, Vs; Figure 2C) of the subpallium also show robust AVT expression. This is also present in Vv, Vd, and the subregions of the Vs (Vsm and Vsl). There is AVT expression in the Vc (Figure 2, C1), and expression is more robust in more caudal regions of the Vs (Vsm, Figure 2, C2). AVT preprohormone mRNA is widely expressed throughout the POA (Figure 3). There is robust expression in parvocellular populations of the POA (Figure 3B), as well as in the magnocellular population (Figure 3C). AVT preprohormone mRNA expression is also present in the gigantocellular population (Figure 3D).

### AVT Expression in the Medial Dorsal Telencephalon

Next, we use qPCR to examine whether AVT preprohormone mRNA expression in the medial dorsal telencephalon is modulated by social context. We find significant variation in AVT expression in the Dm region of the *A. burtoni* telencephalon across social contexts (Figure 4). Specifically, AVT expression is higher in the Familiar Neighbor context as compared to a context with a Neutral Social Stimulus (*p* = 0.003). There is no difference in AVT expression between Reproductive Opportunity context and either Familiar Neighbor or Neutral Social Control contexts.

## Discussion

In the present study, we have shown that expression of AVT preprohormone mRNA in the cichlid fish *A. burtoni* is not limited to preoptic nuclei and the anterior tuberal nucleus. Rather, AVT preprohormone mRNA is expressed widely throughout pallial and subpallial regions not previously associated with the expression of the AVT nonapeptide. We have also found evidence for the social regulation of AVT expression within area Dm-1, the putative homolog of the mammalian basolateral amygdala. These surprising findings provide an important addition to our understanding of the distribution of AVT in the teleost brain and how nonapeptides modulate social behavior in cichlids.

Previous studies in teleost fish have reported the presence of AVT preprohormone and AVT peptide primarily in the POA and the aTn of the hypothalamus (21, 59, 121). Several studies also mapped AVT-immunoreactive fibers and found that they project extensively throughout the teleost brain, although where these fibers originate is not always obvious (55, 121). Our data expand on these studies to show the expression of AVT preprohormone mRNA in multiple regions of the dorsal, medial, central, and ventral pallium. Specifically, subpallial regions, such as the medial and lateral divisions of area Vs [putative homolog of the medial amygdala and the bed nucleus of the stria terminalis (109)] along with area Vv (putative septum homolog) and the central part of area Vd (putative striatum homolog) showed robust expression of AVT preprohormone mRNA, while pallial regions, including basolateral amygdala (area Dm) and hippocampus (area Dl), showed less but still reliably detectable abundance. Our qPCR results confirm expression of AVT in area Dm, and we show this expression to be modulated by the social context. These results suggest that AVT expression in teleosts may be more similar to AVP/T expression in birds and mammals.

If there are indeed AVT expressing neurons in the teleost pallium, why did previous authors fail to detect them? First, methodological limitations may provide an answer: all studies examining the expression and distribution of either AVT preprohormone mRNA or the AVT peptide in teleost fish to date utilize IHC, qPCR, or radioactive ISH to detect peptide and/or mRNA expression (see Table 1). Most do not provide information on telencephalic brain regions, instead focusing exclusively on the preoptic AVT cell populations. The few studies that investigate whether AVT preprohormone or peptide is present in the teleost telencephalon and other areas outside the preoptic nuclei and hypothalamus (21, 51), rely on either radioactive ISH or qPCR of the entire forebrain. Importantly, it is well understood that the former requires short exposure times so as to not overdevelop the signal in preoptic AVT neurons, where the preprohormone is expressed at very high levels [see, e.g., Ref. (21, 59)].

Second, it is also conceivable that AVT transcripts are transported from preoptic cell bodies to fibers (putative axons) in various telencephalic regions for local synthesis (possibly near varicosities or putative release sites). Using both ISH and PCR (122), found oxytocin preprohormone mRNA in axons and Herring bodies in the lateral and ventral hypothalamus, the median eminence, and the posterior lobe of the pituitary in rats. While it is unclear whether this can also occur in axons projecting into the telencephalon, these results nevertheless indicate that at least in the rodent oxytocin preprohormone mRNA can be transported axonally. Given that (a) oxytocin and AVP/T genes as paralogs may share a similar molecular and cellular machinery, and (b) teleosts have brain regions putatively homologous to these rodent regions (109, 117), the signal we detect in pallial regions may indeed be the consequence of axonal transport of AVP/T mRNA. Given the ISH methods used in this study, we cannot conclusively deduce if the mRNA signal resembles varicosities or puncta. Detailed tract tracing studies in combination with sensitive assays such as ISH will allow us to test this hypothesis.

Finally, another possible explanation for the distribution of AVT mRNA expression throughout the teleost telencephalon could be that we are observing preprohormone mRNA that never is translated and processed into the mature peptides AVT and/or neurophysin II. Although the enzymes processing preprohormones could be present in putative pallial AVT neurons for processing peptides others than AVT and neurophysin, any future analysis (e.g., by ISH) demonstrating that these enzymes do not co-localize in these neurons would support this idea. Alternatively, only neurophysin might be produced, for a yet to be discovered function, which can be tested once a specific antibody is available. These possible explanations notwithstanding, our results should be seen as an encouragement to examine telencephalic AVT expression in a range of teleost species.

Is telencephalic AVT of functional importance in *A. burtoni*? Interestingly, we did find significant variation in AVT preprohormone mRNA levels, albeit lowly abundant, depending on social context in area Dm-1, the putative homolog of the mammalian basolateral amygdala (109). This region is known to be important for fear conditioning in mammals, as well as being a sensory integration center that mediates emotional behavior (123, 124). Here, AVT shows increased relative expression in dominant males in the presence of a familiar neighbor, which has important implications for territory defense (125). A possible explanation for this result is that AVT expression in the Dm-1 may be modulating an individual’s behavioral response to a familiar neighbor, possibly facilitating social habituation. It is important to note that we do not know baseline AVT levels in the Dm-1, and the data only represent expression in response to an intruder in a joint defense paradigm (118). Further support for a functional role of AVT expression in the basolateral amygdala homolog is provided by the finding that other candidate genes followed the same expression pattern across experimental groups that we observed with AVT expression, possibly regulated by testosterone (118).

## Conclusion

Nonapeptides are important mediators of social behavior, such as aggression, reproduction, and paternal care, across vertebrates. Their effects are mediated by the presence of receptors and neuronal fibers found throughout the brain. AVP/AVT expression, in particular, has previously been examined across species, and it is canonically held that expression patterns in telencephalic regions of the brain are different between tetrapods and other vertebrates. Previous work has suggested that teleost fish only express AVT cell bodies within the POA-AH complex, and send projections to other telencephalic regions. However, here we find evidence for the presence of AVT preprohormone mRNA in regions previously not associated with AVT expression, such as the dorsomedial, ventral, and central regions of the *A. burtoni* telencephalon. Based on these results, it is worthwhile to reconsider the similarity in AVT/P expression patterns between teleosts and other vertebrates.

## Ethics Statement

The original research reported here was performed under guidelines established and was reviewed and approved by the Institutional Animal Care and Use Committee at The University of Texas at Austin and in compliance with all local, state, and federal regulations.

## Author Contributions

CW, HH, and LH designed the studies; LH conducted the *in situ* hybridization study; CW and JN performed the qPCR experiments; LH, MR-S, and CW performed the data analysis; MR-S and HH wrote the manuscript.

## Conflict of Interest Statement

The authors declare that the research was conducted in the absence of any commercial or financial relationships that could be construed as a potential conflict of interest.

## Abbreviations

AC, anterior commissure; An, anterior thalamic nucleus; aTn, anterior tuberal nucleus; Cn, central nucleus of the inferior lobe; CP, central posterior thalamic nucleus; CV, cerebellar valvula; D, dorsal (pallial) part of the telencephalon; Dc, central part of D; Dc-2, subdivision of Dc; Dd, dorsal part of D; DH, dorsal hypothalamus; Dl, lateral part of D; Dld, dorsal region of Dl; Dlv, ventral region of Dl; Dlvv, ventral zone of Dlv; Dm, medial part of D; Dm-1,2,3, subdivisions of Dm; Dm2c, caudal part of Dm-2; Dn, diffuse nucleus of the inferior lobe; Dp, posterior part of D; Dx, unassigned part of D; E, entopeduncular nucleus; GR, corpus glomerulosum pars rotunda; H, habenula; HC, horizontal commissure; IL, inferior lobe; LHn, lateral hypothalamic nucleus; LPGn, lateral preglomerular nucleus; LR, lateral recess; LT, longitudinal torus; LZ, zona limitans of the diencephalon; MB, mammillary body; mPGn, medial preglomerular nucleus; nLT, nucleus of the lateral torus; nMLF, nucleus of the medial longitudinal fascicle; OB, olfactory bulb; OPT, optic tract; OT, optic tectum; P, pituitary; PAG, periaqueductal gray; PGCn, preglomerula commissural nucleus; PN, prethalamic nucleus; POA, preoptic area; PPd, dorsal periventricular pretectal nucleus; PPr, rostral periventricular pretectal nucleus; pTGN, preglomerular tertiary gustatory nucleus; pTn, posterior tuberal nucleus; PVO, paraventricular organ; ST, semicircular torus; TPp, periventricular nucleus of the posterior tuberculum; V, ventral (subpallial) division of the telencephalon; Vc, central part of V; Vd, dorsal part of V; Vdc, caudal region of Vd; Vdr, rostral region of Vd; VH, ventral hypothalamus; Vi, intermediate part of V; Vl, lateral part of V; VM, ventromedial thalamic nucleus; Vp, postcommissural nucleus of V; vPPn, ventral portion of the periventricular pretectal nucleus; Vs, supracommissural nucleus of V; Vsl, lateral region of Vs; Vsm, medial region of Vs; vTn, ventral tuberal nucleus; Vv, ventral part of V.

## References

[1] Goodson JL Thompson RR. Nonapeptide mechanisms of social cognition, behavior and species-specific social systems. Curr Opin Neurobiol (2010) 20:784–94. 10.1016/j.conb.2010.08.020 20850965

[2] Goodson JL Deconstructing sociality, social evolution and relevant nonapeptide functions. Psychoneuroendocrinology (2012) 38:465–78. 10.1016/j.psyneuen.2012.12.005 23290368

[3] Goodson JL Bass AH. Social behavior functions and related anatomical characteristics of vasotocin/vasopressin systems in vertebrates. Brain Res Rev (2001) 35:246–65. 10.1016/S0165-0173(01)00043-1 11423156

[4] Pang PKT Furspand PB Sawyer WH Evolution of neurohypophysial hormone actions in vertebrates. Am Zool (1983) 23:655–62. 10.1093/icb/23.3.655

[5] George JC Comparative physiology of metabolic responses to neurohypophysial hormones in vertebrates. Am Zool (1977) 17:788–808. 10.1093/icb/17.4.787

[6] Viau V Sharma S Plotsky P Meaney MJ. Increased plasma ACTH responses to stress in nonhandled compared with handled rats require basal levels of corticosterone and are associated with increased levels of ACTH secretagogues in the median eminence. J Neurosci (1993) 13:1097–105. 8382733 10.1523/JNEUROSCI.13-03-01097.1993 PMC6576607

[7] Salek SJ Sullivan CV Godwin J. Arginine vasotocin effects on courtship behavior in male white perch (*Morone americana*). Behav Brain Res (2002) 133:177–83. 10.1016/S0166-4328(02)00003-7 12110451

[8] Thompson RR Walton JC Peptide effects on social behavior: effects of vasotocin and isotocin on social approach behavior in male goldfish (*Carassius auratus*). Behav Neurosci (2004) 118:620–6. 10.1037/0735-7044.118.3.620 15174940

[9] Santangelo N Bass A Individual behavioral and neuronal phenotypes for arginine vasotocin mediated courtship and aggression in a territorial teleost. Brain Behav Evol (2010) 75:282–91. 10.1159/000316867 20693783

[10] Moore FL Miller LJ. Arginine vasotocin induces sexual behavior of newts by acting on cells in the brain. Peptides (1983) 4:97–102. 10.1016/0196-9781(83)90173-0 6866813

[11] Boyd SK. Arginine vasotocin facilitation of advertisement calling and call phonotaxis in bullfrogs. Horm Behav (1994) 28:232–40. 10.1006/hbeh.1994.1020 7814004

[12] Marler CA Chu J Wilczynski W. Arginine vasotocin injection increases probability of calling in cricket frogs, but causes call changes characteristic of less aggressive males. Horm Behav (1995) 29:554–70. 10.1006/hbeh.1995.1286 8748513

[13] de Kloet ER Elands J Voorhuis DAM Implication of central neurohypophyseal hormone receptor-mediated action in the timing of reproductive events: evidence from novel observations on the effect of a vasotocin analogue on singing behavior of the canary. Regul Pept (1993) 45:85–9. 10.1016/0167-0115(93)90187-D 8390083

[14] Maney DL Goode CT Wingfield JC. Intraventricular infusion of arginine vasotocin induces singing in a female songbird. J Neuroendocrinol (1997) 9:487–91. 10.1046/j.1365-2826.1997.00635.x 15305566

[15] Goodson JL Territorial aggression and dawn song are modulated by septal vasotocin and vasoactive intestinal polypeptide in male field sparrows (*Spizella pusilla*). Horm Behav (1998) 34:67–77. 10.1006/hbeh.1998.1467 9735230

[16] Goodson JL Vasotocin and vasoactive intestinal polypeptide modulate aggression in a territorial songbird, the violet-eared waxbill (Estrildidae: *Uraeginthus granatina*). Gen Comp Endocrinol (1998) 111:233–44. 10.1006/gcen.1998.7112 9679095

[17] Ferris CF Delville Y. Vasopressin and serotonin interactions in the control of agonistic behavior. Psychoneuroendocrinology (1994) 19:593–601. 10.1016/0306-4530(94)90043-4 7938357

[18] Albers HE Bamshad M Role of vasopressin and oxytocin in the control of social behavior in Syrian hamsters (*Mesocricetus auratus*). Prog Brain Res (1999) 119:395–408. 10.1016/S0079-6123(08)61583-6 10074802

[19] Wang Z Young LJ De Vries GJ Insel TR Voles and vasopressin: a review of molecular, cellular, and behavioral studies of pair bonding and paternal behaviors. Prog Brain Res (1999) 119:483–99. 10.1016/S0079-6123(08)61589-7 10074808

[20] Oldfield RG Harris RM Hofmann HA Integrating resource defence theory with a neural nonapeptide pathway to explain territory-based mating systems. Front Zool (2015) 12:S16 10.1186/1742-9994-12-S1-S16 26813803 PMC4722349

[21] Greenwood AK Wark AR Fernald RD Hofmann HA Expression of arginine vasotocin in distinct preoptic regions is associated with dominant and subordinate behavior in an African cichlid fish. Proc Biol Sci (2008) 275:2393–402. 10.1098/rspb.2008.0622 18628117 PMC2603226

[22] Goodson J Bass A Forebrain peptides modulate sexually polymorphic vocal circuitry. Nature (2000) 403:769–72. 10.1038/35001581 10693805

[23] Goodson J Bass A Vasotocin innervation and modulation of vocal-acoustic circuitry in the teleost *Porichthys notatus*. JComp Neurol (2000) 422:363–79. 10.1002/1096-9861(20000703)422:3<363::AID-CNE4>3.0.CO;2-8 10861513

[24] Semsar K Kandel F Godwin J. Manipulations of the AVT system shift social status and related courtship and aggressive behavior in the bluehead wrasse. Horm Behav (2001) 40:21–31. 10.1006/hbeh.2001.1663 11467881

[25] Foran CM Bass AH. Preoptic GnRH and AVT: axes for sexual plasticity in teleost fish. Gen Comp Endocrinol (1999) 116:141–52. 10.1006/gcen.1999.7357 10562445

[26] Grober MS George AA Watkins KK Carneiro LA Oliveira RF. Forebrain AVT and courtship in a fish with male alternative reproductive tactics. Brain Res Bull (2002) 57:423–5. 10.1016/S0361-9230(01)00704-3 11923002

[27] Miranda JA Oliveira RF Carneiro LA Santos RS Grober MS. Neurochemical correlates of male polymorphism and alternative reproductive tactics in the Azorean rock-pool blenny, *Parablennius parvicornis*. Gen Comp Endocrinol (2003) 132:183–9. 10.1016/S0016-6480(03)00063-7 12812764

[28] Ota Y Ando H Ueda H Urano A. Differences in seasonal expression of neurohypophysial hormone genes in ordinary and precocious male masu salmon. Gen Comp Endocrinol (1999) 116:40–8. 10.1006/gcen.1999.7343 10525360

[29] Larson ET O’Malley DM Melloni RH Jr. Aggression and vasotocin are associated with dominant-subordinate relationships in zebrafish. Behav Brain Res (2006) 167:94–102. 10.1016/j.bbr.2005.08.020 16213035

[30] Robinson A Neurophysins, an aid to understanding the neurohypophysis. Front Neuroendocrinol (1978) 5:35–59.

[31] Goodson JL Nonapeptides and evolutionary patterning of sociality. Prog Brain Res (2008) 170:3–15. 10.1016/S0079-6123(08)00401-9 18655867 PMC2570786

[32] Baulieu EE Kelly PA Hormones: From Molecules to Disease. New York: Springer Science+Business Media (1990).

[33] Balment R Lu W Weybourne E Warne JM Arginine vasotocin a key hormone in fish physiology and behavior: a review with insights from mammalian models. Gen Comp Endocrinol (2006) 147:9–16. 10.1016/j.ygcen.2005.12.022 16480986

[34] Kline R Holt G Khan I Arginine vasotocin V1a2 receptor and GnRH-I colocalize in preoptic neurons of the sex changing grouper, *Epinephelus adscensionis*. Gen Comp Endocrinol (2015) 225:33–44. 10.1016/j.ygcen.2015.07.013 26361870

[35] Insel TR Wang Z Ferris CF. Patterns of brain vasopressin receptor distribution associated with social organization in microtine rodents. J Neurosci (1994) 14:5381–92. 8083743 10.1523/JNEUROSCI.14-09-05381.1994 PMC6577077

[36] Goodson JL Wang Y. Valence-sensitive neurons exhibit divergent functional profiles in gregarious and asocial species. Proc Natl Acad Sci U S A (2006) 103:17013–7. 10.1073/pnas.0606278103 17071744 PMC1636570

[37] Baeyens DA Cornett LE The cloned avian neurohypophysial hormone receptor. Comp Biochem Physiol B Biochem Mol Biol (2006) 143:12–9. 10.1016/j.cbpb.2005.09.012 16311051

[38] Konno N Hyodo S Yamaguchi Y Kaiya H Miyazato M Matsuda K African lungfish, *Protopterus annectens*, possess an arginine vasotocin receptor homologous to the tetrapod V2-type receptor. J Exp Biol (2009) 212:2183–93. 10.1242/jeb.029322 19561208

[39] Konno N Kurosawa M Kaiya H Miyazato M Matsuda K Uchiyama M. Molecular cloning and characterization of V2-type receptor in two ray-finned fish, gray bichir, *Polypterus senegalus*, and medaka, *Oryzas latipes*. Peptides (2010) 31:1273–9. 10.1016/j.peptides.2010.04.014 20420873

[40] Lema S. Identification of multiple vasotocin receptor cDNAs in teleost fish: sequences, phylogenetic analysis, sites of expression, and regulation in the hypothalamus and gill in response to hyperosmotic challenge. Mol Cell Endocrinol (2010) 321:215–30. 10.1016/j.mce.2010.02.015 20167249

[41] Kline R O’Connell L Hofmann HA Holt GJ Khan IA. The distribution of an AVT V1a receptor in the brain of a sex changing fish, *Epinephelus adscensionis*. J Chem Neuroanat (2011) 42:72–88. 10.1016/j.jchemneu.2011.06.005 21723386

[42] Huffman LS O’Connell LA Kenkel CD Kline RJ Khan IA Hofmann HA. Distribution of nonapeptide systems in the forebrain of an African cichlid fish, *Astatotilapia burtoni*. J Chem Neuroanat (2012) 44:86–97. 10.1016/j.jchemneu.2012.05.002 22668656

[43] de Vries GJ Miller MA Anatomy and function of extrahypothalamic vasopressin systems in the brain. Prog Brain Res (1999) 119:3–20. 10.1016/S0079-6123(08)61558-7 10074777

[44] Engelmann M Wotjak CT Neumann I Ludwig M Landgraf R. Behavioral consequences of intracerebral vasopressin and oxytocin: focus on learning and memory. Science (1996) 20:341–58. 8880728 10.1016/0149-7634(95)00059-3

[45] Lowry CA Richardson CF Zoeller TR Miller LJ Muske LE Moore FL Neuroanatomical distribution of vasotocin in a urodele amphibian (*Taricha granulosa*) revealed by immunohistochemical and *in situ* hybridization techniques. JComp Neurol (1997) 385:43–70. 10.1002/(SICI)1096-9861(19970818)385:1<43::AID-CNE3>3.0.CO;2-C 9268116

[46] Moore FL. Evolutionary precedents for behavioral actions of oxytocin and vasopressin. Ann N Y Acad Sci (1992) 652:156–65. 10.1111/j.1749-6632.1992.tb34352.x 1626827

[47] Moore FL Lowry CA. Comparative neuroanatomy of vasotocin and vasopressin in amphibians and other vertebrates. Comp Biochem Physiol C (1998) 199:251–60. 9826998 10.1016/s0742-8413(98)00014-0

[48] Winslow J Insel TR. Vasopressin modulates male squirrel monkeys’ behavior during social separation. Eur J Pharmacol (1991) 200:95–101. 10.1016/0014-2999(91)90671-C 1769376

[49] Olivereau M Moons L Olivereau J Vandesande F. Coexistence of corticotropin-releasing factor-like immunoreactivity and vasotocin in perikarya of the preoptic nucleus in the eel. Gen Comp Endocrinol (1988) 70:41–8. 10.1016/0016-6480(88)90092-5 3286370

[50] Reaves TA Hayward JN. Functional and morphological studies of peptide-containing neuroendocrine cells in goldfish hypothalamus. J Comp Neurol (1980) 193:777–88. 10.1002/cne.901930313 7440790

[51] Hur S Takeuchi Y Esaka Y Nina W Park Y Kang H Diurnal expression patterns of neurohypophysial hormone genes in the brain of the threespot wrasse *Halichoeres trimaculatus*. Comp Biochem Physiol A Mol Integr Physiol (2011) 158:490–7. 10.1016/j.cbpa.2010.12.011 21167953

[52] Ota Y Ando H Ueda H Urano A Sexually different expression of neurohypophysial hormone genes in the preoptic nucleus of pre-spawning chum salmon. Zoolog Sci (1996) 13:593–601. 10.2108/zsj.13.593

[53] Ota Y Ando H Ueda H Urano A Seasonal changes in expression of neurohypophysial hormone genes in the preoptic nucleus of immature female masu salmon. Gen Comp Endocrinol (1999) 116:31–9. 10.1006/gcen.1999.7343 10525359

[54] Gilchriest BJ Tipping DR Hake L Levy A Baker BI. The effects of acute and chronic stresses on vasotocin gene transcripts in the brain of the rainbow trout (*Oncorhynchus mykiss*). J Neuroendocrinol (2000) 12:795–801. 10.1046/j.1365-2826.2000.00522.x 10929092

[55] Batten TFC Cambre ML Moons L Vandesande F. Comparative distribution of neuropeptide-immunoreactive systems in the brain of the green molly *Poecilia latipinna*. J Comp Neurol (1990) 302:893–919. 10.1002/cne.903020416 2081820

[56] Goossens N Dierickx K Vandesande F. Immunocytochemical study of the neurohypophysial hormone producing system of the lungfish, *Protopterus aethiopicus*. Cell Tissue Res (1978) 190:69–77. 10.1007/BF00210037 357002

[57] van den Dungen HM Buijs RM Pool CW Terlou M. The distribution of vasotocin and isotocin in the brain of the rainbow trout. J Comp Neurol (1982) 212:146–57. 10.1002/cne.902120205 6765094

[58] Vallarino M Viglietti-Panzica C Panzica GC Immunocytochemical localization of vasotocin-like immunoreactivity in the brain of the cartilaginous fish *Scyliorhinus caniculus*. Cell Tissue Res (1990) 262:507–14. 10.1007/BF00305246

[59] Godwin J Sawby R Warner RR Crews D Grober MS. Hypothalamic arginine vasotocin mRNA abundance variation across sexes and with sex change in a coral reef fish. Brain Behav Evol (2000) 55:77–84. 10.1159/000006643 10838478

[60] Schreibman MP Halpern LR The demonstration of neurophysin and arginine vasotocin by immunocytochemical methods in the brain and pituitary of the platyfish, *Xiphophorus maculatus*. Gen Comp Endocrinol (1980) 40:1–7. 10.1016/0016-6480(80)90089-1 7353780

[61] Gonzalez A Smeets WJAJ. Comparative analysis of the vasotocinergic and mesotocinergic cells and fibers in the brain of two amphibians, the anuran *Rana ridibunda* and the urodele *Pleurodeles waltlii*. J Comp Neurol (1992) 315:53–73. 10.1002/cne.903150105 1541723

[62] Gonzalez A Smeets WJAJ Distribution of vasotocin- and mesotocin-like immunoreactivities in the brain of the South African clawed frog *Xenopus laevis*. J Chem Neuroanat (1992) 32:371–5. 10.1016/0891-0618(92)90003-9 1476666

[63] Boyd SK Tyler CJ De Vries GJ. Sexual dimorphism in the vasotocin system of the bullfrog (*Rana catesbeiana*). J Comp Neurol (1992) 325:313–25. 10.1002/cne.903250213 1460117

[64] Mathieson WB. Development of arginine vasotocin innervation in two species of anuran amphibian, *Rana catesbeiana* and *Rana sylvatica*. Histochem Cell Biol (1996) 105:305–18. 10.1007/BF01463933 9072187

[65] Lowry CA Renner KJ Moore FL. Catecholamines and indoleamines in the central nervous system of a urodele amphibian: a microdissection study with emphasis on the distribution of epinephrine. Brain Behav Evol (1996) 48:70–93. 10.1159/000113187 8853874

[66] Jokura Y Urano A Extrahypothalamic projection of immunoreactive vasotocin fibers in the brain of the toad, *Bufo japonicas*. Zool Sci (1987) 4:675–81.

[67] Smeets WJAJ Sevensma JJ Jonker AJ. Comparative analysis of vasotocin-like immunoreactivity in the brain of the turtle *Pseudemys scripta elegans* and the snake *Python regius*. Brain Behav Evol (1990) 35:65–84. 10.1159/000115857 2191754

[68] Gonzalez A Smeets WJAJ Distribution of vasotocin- and mesotocin-like immunoreactivities in the brain of *Typhlonectes compressicauda* (Amphibia, Gymnophiona): further assessment of primitive and derived traits of amphibian neuropeptidergic systems. Cell Tissue Res (1997) 287:305–14. 10.1007/s004410050755 8995201

[69] Hilscher-Conklin C Conlon JM Boyd SK Identification and localization of neurohypophysial peptides in the brain of a caecilian amphibian, *Typhlonectes natans* (Amphibia: Gymnophiona). J Comp Neurol (1998) 394:139–51. 10.1002/(SICI)1096-9861(19980504)394:2<139::AID-CNE1>3.3.CO;2-A 9552122

[70] Propper CR Jones RE Lopez KH. Distribution of arginine vasotocin in the brain of the lizard *Anolis carolinensis*. Cell Tissue Res (1992) 267:391–8. 10.1007/BF00302978 1600566

[71] Smeets WJAJ Sevensma JJ Jonker AJ Blahser S Comparative analysis of vasotocin-like immunoreactivity in the brain of the turtle *Pseudemys scripta elegans* and the snake *Python regius*. Brain Behav Evol (1983) 35:11–24. 10.1159/000115857 2191754

[72] Stoll CJ Voorn P. The distribution of hypothalamic and extrahypothalamic vasotocinergic cells and fibers in the brain of a lizard, *Gekko gecko*: presence of a sex difference. J Comp Neurol (1985) 239:193–204. 10.1002/cne.902390206 4044934

[73] Thepen T Voorn P Stoll CJ Sluiter AA Pool CW Lohman AH. Mesotocin and vasotocin in the brain of the lizard *Gekko gecko*: an immunocytochemical study. Cell Tissue Res (1987) 250:649–56. 10.1007/BF00218959 3690641

[74] Bons N. Immunocytochemical identification of the mesotocin- and vasotocin-producing systems in the brain of temperate and desert lizard species and their modifications by cold exposure. Gen Comp Endocrinol (1983) 52:56–66. 10.1016/0016-6480(83)90158-2 6628979

[75] Fernandez-Llebrez P Perez J Nadales AE Cifuentes M Grondona JM Mancera JM Immunocytochemical study of the hypothalamic magnocellular neurosecretory nuclei of the snake *Natrix maura* and the turtle *Mauremys caspica*. Cell Tissue Res (1988) 253:435–45. 10.1007/BF00222301 3409295

[76] Aste N Muhlbauer E Grossmann R. Distribution of AVT gene expressing neurons in the prosencephalon of Japanese quail and chicken. Cell Tissue Res (1996) 286:365–73. 10.1007/s004410050706 8929339

[77] Jurkevich A Barth SW Aste N Panzica GC Grossmann R. Intracerebral sex differences in the vasotocin system in birds: possible implication in behavioral and autonomic functions. Horm Behav (1996) 30:673–81. 10.1006/hbeh.1996.0068 9047289

[78] Panzica GC Plumari L Garcia-Ojeda E Deviche P. Central vasotocin-immunoreactive system in a male passerine bird (*Junco hyemalis*). JComp Neurol (1999) 409:105–17. 10.1002/(SICI)1096-9861(19990621)409:1<105::AID-CNE8>3.0.CO;2-8 10363714

[79] Kiss JZ Voorhuis TA van Eekelen JA de Kloet ER de Wied D. Organization of vasotocin-immunoreactive cells and fibers in the canary brain. J Comp Neurol (1987) 263:347–64. 10.1002/cne.902630304 3667983

[80] Voorhuis TAM de Kloet ER. Immunoreactive vasotocin in the zebra finch brain (*Taeniopygia guttata*). Brain Res Dev Brain Res (1992) 69:1–10. 10.1016/0165-3806(92)90116-E 1424081

[81] Berk ML Reaves TA Hayward JN Finkelstein JA. The localization of vasotocin and neurophysin neurons in the diencephalon of the pigeon, Columba livia. J Comp Neurol (1982) 204:392–406. 10.1002/cne.902040410 7061740

[82] Bons N. The topography of mesotocin and vasotocin systems in the brain of the domestic mallard and Japanese quail: immunocytochemical identification. Cell Tissue Res (1980) 213:37–51. 10.1007/BF00236919 7459995

[83] Panzica GC Calcagni M Ramieri G Viglietti-Panzica C. Extrahypothalamic distribution of vasotocin-immunoreactive fibers and perikarya in the avian central nervous system. Basic Appl Histochem (1988) 32:89–94. 3390126

[84] Caverson MM Ciriello J Calaresu FR Krukoff TL. Distribution and morphology of vasopressin-, neurophysin II-, and oxytocin-immunoreactive cell bodies in the forebrain of the cat. J Comp Neurol (1987) 259:211–36. 10.1002/cne.902590204 3294931

[85] Caffe AR Van Ryen PC Vand Der Woude TP van Leeuwen FW. Vasopressin and oxytocin systems in the brain and upper spinal cord of *Macaca fascicularis*. J Comp Neurol (1989) 287:302–25. 10.1002/cne.902870304 2778107

[86] Dubois-Dauphin M Tribollet E Dreifuss JJ Distribution of neurohypophysial peptides in the guinea pig brain. I. An immunocytochemical study of the vasopressin-related glycopeptide. Brain Res (1990) 496:45–65. 10.1016/0006-8993(89)91051-2 2804653

[87] Castel M Morris JF. The neurophysin-containing innervation of the forebrain of the mouse. Neuroscience (1988) 24:937–66. 10.1016/0306-4522(88)90078-4 3380308

[88] Rhodes CH Morrell JI Pfaff DW. Immunohistochemical analysis of magnocellular elements in rat hypothalamus: distribution and numbers of cells containing neurophysin, oxytocin, and vasopressin. J Comp Neurol (1981) 198:45–64. 10.1002/cne.901980106 7014660

[89] DeVries GJ Buijs RM van Leeuwen FW Caffe AR Swaab DF. The vasopressinergic innervation of the brain in normal and castrated rats. J Comp Neurol (1985) 233:236–54. 10.1002/cne.902330206 3882778

[90] van Leeuwen FW Caffe AR de Vries GJ. Vasopressin cells in the bed nucleus of the stria terminalis of the rat: sex differences and the influence of androgens. Brain Res (1985) 325:391–4. 10.1016/0006-8993(85)90348-8 3978433

[91] Urban JH Miller MA Drake CT Dorsa DM Detection of vasopressin mRNA in cells of the medial amygdala but not in the locus coeruleus by *in situ* hybridization. J Chem Neuroanat (1990) 3:277–83. 2397053

[92] Wang Z Ferris CF de Vries GJ. Role of septal vasopressin innervation in paternal behavior in prairie voles (*Microtus ochrogaster*). Proc Natl Acad Sci U S A (1994) 91:400–4. 10.1073/pnas.91.1.400 8278401 PMC42955

[93] Planas B Kolb PE Rasking MA Miller MA. Vasopressin and galanin mRNAs coexist in the nucleus of the horizontal diagonal band: a novel site of vasopressin gene expression. J Comp Neurol (1995) 361:48–56. 10.1002/cne.903610105 8550881

[94] van Eerdenburg FJCM Swaab DF van Leeuwen FW. Distribution of vasopressin and oxytocin cells and fibres in the hypothalamus of the domestic pig (*Sus scrofa*). J Comp Neurol (1992) 318:138–46. 10.1002/cne.903180203 1583158

[95] Lakhdar-Ghazal N Dubois-Dauphin M Hermes MLHJ Buijs RM Bengelloun WA Pevet P. Vasopressin in the brain of a desert hibernator, the jerboa (*Jaculus orientalis*): presence of sexual dimorphism and seasonal variation. J Comp Neurol (1995) 358:499–517. 10.1002/cne.903580404 7593745

[96] Wu CM Shen CL The distribution of vasopressinergic and oxytocinergic neurons in the CNS of the gerbil. Zool Studies (1994) 33:114–25.

[97] Dobie DJ Miller MA Raskind MA Dorsa DM. Testosterone reverses a senescent decline in extrahypothalamic vasopressin mRNA. Brain Res (1992) 583:247–52. 10.1016/S0006-8993(10)80030-7 1504830

[98] Miller MA Urban JH Dorsa DM. Steroid dependency of vasopressin neurons in the bed nucleus of the stria terminalis by in situ hybridization. Endocrinology (1989) 125:2335–40. 10.1210/endo-125-5-2335 2791993

[99] Miller MA De Vries GJ al-Shamma HA Dorsa DM Decline of vasopressin immunoreactivity and mRNA levels in the bed nucleus of the stria terminalis following castration. J Neurosci (1992) 12:2881–7. 1494938 10.1523/JNEUROSCI.12-08-02881.1992 PMC6575668

[100] Brot MD Bernstein IL Dorsa DM. Vasopressin deficiency abolishes a sexually dimorphic behavior in Brattleboro rats. Physiol Behav (1992) 51:839–43. 10.1016/0031-9384(92)90124-K 1594683

[101] Szot P Dorsa DM. Differential timing and sexual dimorphism in the expression of the vasopressin gene in the developing rat brain. Brain Res Dev Brain Res (1993) 73:177–83. 10.1016/0165-3806(93)90136-X 8353930

[102] Szot P Dorsa DM. Expression of cytoplasmic and nuclear vasopressin RNA following castration and testosterone replacement: evidence for transcriptional regulation. Mol Cell Neurosci (1994) 5:1–10. 10.1006/mcne.1994.1001 8087411

[103] DeVries GJ Buijs RM. The origin of the vasopressinergic and oxytocinergic innervation of the rat brain; with special reference to the lateral septum. Brain Res (1983) 273:307–17. 10.1016/0006-8993(83)90855-7 6311351

[104] Caffe AR Van Leeuwen FW Luiten PG. Vasopressin cells in the medial amygdala of the rat project to the lateral septum and ventral hippocampus. J Comp Neurol (1987) 261:237–52. 10.1002/cne.902610206 3305600

[105] Winslow JT Hastings N Carter CS Harbaugh CR Insel TR. A role for central vasopressin in pair bonding in monogamous prairie voles. Nature (1993) 365:545–8. 10.1038/365545a0 8413608

[106] Young LJ Nilsen R Waymire KG MacGregor GR Insel TR. Increased affiliative response to vasopressin in mice expressing the V1a receptor from a monogamous vole. Nature (1999) 400:766–8. 10.1038/23475 10466725

[107] Liu Y Curtis T Wang Z. Vasopressin in the lateral septum regulates pair bond formation in male prairie voles (*Microtus ochrogaster*). Behav Neurosci (2001) 115:910–9. 10.1037/0735-7044.115.4.910 11508730

[108] Kapsimali M Bourrat F Vernier P Distribution of the orphan nuclear receptor Nurr1 in medaka (*Oryzias latipes*): cues to the definition of homologous cell groups in the vertebrate brain. J Comp Neurol (2001) 292:276–92. 10.1002/1096-9861(20010312)431:3<276::AID-CNE1070>3.0.CO;2-S 11170005

[109] O’Connell LA Hofmann HA. The vertebrate mesolimbic reward system and social behavior network: a comparative synthesis. Journal of Comparative Neurology (2011) 519:3599–3639. 10.1002/cne.22735 21800319

[110] Goodson JL. The vertebrate social behavior network: evolutionary themes and variations. Horm Behav (2005) 48:11–22. 10.1016/j.yhbeh.2005.02.003 15885690 PMC2570781

[111] Forlano PM Bass AH. Neural and hormonal mechanisms of reproductive-related arousal in fishes. Horm Behav (2011) 59:616–29. 10.1016/j.yhbeh.2010.10.006 20950618 PMC3033489

[112] Saito D Komatsuda M Urano A. Functional organization of preoptic vasotocin and isotocin neurons in the brain of rainbow trout: central and neurohypophysial projections of single neurons. Neuroscience (2004) 124:973–84. 10.1016/j.neuroscience.2003.12.038 15026137

[113] Gilchriest BJ Tipping DR Levy A Baker BI. Diurnal changes in the expression of genes encoding for arginine vasotocin and pituitary pro-opiomelanocortin in the rainbow trout (*Oncorhynchus mykiss*): correlation with changes in plasma hormones. J Neuroendocrinol (1998) 10:937–43. 10.1046/j.1365-2826.1998.00283.x 9870751

[114] Young LJ Wang ZX. The neurobiology of pair bonding. Nat Neurosci (2004) 7:1048–54. 10.1038/nn1327 15452576

[115] Maney DL Erwin KL Goode CT. Neuroendocrine correlates of behavioral polymorphism in white-throated sparrows. Horm Behav (2005) 48:196–206. 10.1016/j.yhbeh.2005.03.004 15878570

[116] Munchrath LA Hofmann HA. Distribution of sex steroid hormone receptors in the brain of an African cichlid fish, *Astatotilapia burtoni*. J Comp Neurol (2010) 518:3302–26. 10.1002/cne.22401 20575061

[117] O’Connell LA Hofmann HA. Social status predicts how sex steroid receptors regulate complex behavior across levels of biological organization. Endocrinology (2012) 153:1341–51. 10.1210/en.2011-1663 22166981

[118] Weitekamp C Nguyen J Hofmann HA Social context affects behavior, preoptic area gene expression, and response to D2 receptor manipulation during territorial defense in a cichlid fish. Genes Brain Behav (2017) 16: 601–11. 10.1111/gbb.12389 28466980

[119] Kidd MR Dijkstra PD Alcott C Lavee D Ma J O’Connell LA Prostaglandin F2α facilitates female mating behavior based on male performance. Behav Ecol Sociobiol (2013) 67:1307–15. 10.1007/s00265-013-1559-919561208

[120] Matz MV Wright RM Scott JG. No control genes required: Bayesian analysis of qRT-PCR data. PLoS One (2013) 8:e71448. 10.1371/journal.pone.0071448 23977043 PMC3747227

[121] Goodson JL Evans AK Bass AH. Putative isotocin distributions in sonic fish: relation to vasotocin and vocal-acoustic circuitry. JComp Neurol (2003) 462:1–14. 10.1002/cne.10679 12761820 PMC2679688

[122] Jirikowski GF Sanna PP Bloom FE. mRNA coding for oxytocin is present in axons of the hypothalamo-neurohypophysial tract. Proc Natl Acad Sci U S A (1990) 87:7400–4. 10.1073/pnas.87.24.10068c 2268384 PMC54754

[123] LeDoux JE Emotion circuits in the brain. Annu Rev Neurosci (2000) 23:155–84. 10.1146/annurev.neuro.23.1.155 10845062

[124] Moreno N Gonzalez A. Evolution of the amygdaloid complex in vertebrates, with special reference to the anamnio-amniotic transition. J Anat (2007) 211:151–63. 10.1111/j.1469-7580.2007.00780.x 17634058 PMC2375767

[125] Weitekamp C Hofmann HA. Neuromolecular correlates of cooperation and conflict during territory defense in a cichlid fish. Horm Behav (2017) 89:145–56. 10.1016/j.yhbeh.2017.01.001 28108326

[126] Greenwood AK Plasticity in the Neural Control of Reproductive Behavior and Physiology [Doctoral thesis]. Palo Alto (CA): Stanford University (2004).

